# The etiology of idiopathic congenital talipes equinovarus: a systematic review

**DOI:** 10.1186/s13018-018-0913-z

**Published:** 2018-08-22

**Authors:** Vito Pavone, Emanuele Chisari, Andrea Vescio, Ludovico Lucenti, Giuseppe Sessa, Gianluca Testa

**Affiliations:** Department of General Surgery and Medical Surgical Specialties, Section of Orthopaedics and Traumatology, University Hospital Policlinico-Vittorio Emanuele, University of Catania, Via Plebiscito, 628, 95124 Catania, Italy

**Keywords:** Clubfoot, ICTEV, Pathogenesis, Genetics, Risk factors, Etiology

## Abstract

**Background:**

Also known as clubfoot, idiopathic congenital talipes equinovarus (ICTEV) is the most common pediatric deformity and occurs in 1 in every 1000 live births. Even though it has been widely researched, the etiology of ICTEV remains poorly understood and is often described as being based on a multifactorial genesis. Genetic and environmental factors seem to have a major role in the development of this disease. Thus, the aim of this review is to analyze the available literature to document the current evidence on ICTEV etiology.

**Methods:**

The literature on ICTEV etiology was systematically reviewed using the following inclusion criteria: studies of any level of evidence, reporting clinical or preclinical results, published in the last 20 years (1998–2018), and dealing with the etiology of ICTEV.

**Results:**

A total of 48 articles were included. ICTEV etiology is still controversial. Several hypotheses have been researched, but none of them are decisive. Emerging evidence suggests a role of several pathways and gene families associated with limb development (HOX family; PITX1-TBX4), the apoptotic pathway (caspases), and muscle contractile protein (troponin and tropomyosin), but a major candidate gene has still not been identified. Strong recent evidence emerging from twin studies confirmed major roles of genetics and the environment in the disease pathogenesis.

**Conclusions:**

The available literature on the etiology of ICTEV presents major limitations in terms of great heterogeneity and a lack of high-profile studies. Although many studies focus on the genetic background of the disease, there is lack of consensus on one or multiple targets. Genetics and smoking seem to be strongly associated with ICTEV etiology, but more studies are needed to understand the complex and multifactorial genesis of this common congenital lower-limb disease.

## Background

Congenital talipes equinovarus (CTEV) is a foot deformity characterized by hindfoot varus, forefoot (metatarsus) adductus, an augmented midfoot arch (cavus), and equinus. This pediatric malformation can be classified according to its clinical presentation. It can be secondary or syndromic when its presentation is associated with another congenital disease (20% of cases). However, it may also occur as an isolated birth defect with no other malformations (80% of cases), which introduces the concept of idiopathic CTEV (ICTEV). The etiology of CTEV is largely unknown. Secondary CTEV is usually a manifestation of distal arthrogryposis (DA), congenital myotonic dystrophy, myelomeningocele, or other congenital diseases. While the clinical presentation may be similar to the idiopathic form, secondary CTEV seems to derive from neuromuscular [1] and fetal abnormalities [2] involved in its etiopathogenesis, thus making ICTEV and syndromic CTEV rather different in clinical presentation, treatment, and proposed etiopathogenetic mechanism [3, 4].

ICTEV is one of the most common pediatric deformities. The epidemiological studies published over the last 55 years suggest a birth prevalence in the range of 0.5 to 2.0 cases/1000 live births, which results in an estimated 7–43 cases of clubfoot/year/million population, depending mainly on the birth rate [5]. The higher prevalence seems to be associated with social-demographic, genetic, and environmental risk factors, which explain its prevalence among low- and middle-income countries [5] and closed societies like the Maori population [6]. It affects males more than females [7] with a male-to-female ratio of 2:1, which is similar across different ethnic groups [8–11]. Kruse et al. proposed a reason for the gender difference in 2008 [12], but the phenotypic variability in affected individuals is still unknown.

Several treatments have been proposed throughout the centuries, but today, the gold-standard treatment is the Ponseti method [13, 14]. In syndromic cases, current evidence supports the Ponseti method or other more invasive surgical procedures [15]. The aim of this review is to analyze the available literature to provide an update on the evidence related to ICTEV etiology.

## Materials and methods

We conducted this systematic review according to the guidelines of the Preferred Reporting Items for Systematic Reviews and Meta-Analyses (PRISMA) [16]. Two medical electronic databases (PubMed and Science Direct) were searched by a single author (CE) on March 20, 2018. The research string used was “(clubfoot OR congenital talipes equinovarus OR clubfeet) AND (pathology OR embryology OR etiology OR etiopathogenesis OR genetics OR pathophysiology).” A total of *n* = 1590 articles were found. After excluding duplicates, *n* = 974 articles were selected.

The initial titles and abstracts were screened using the following inclusion criteria: studies of any level of evidence reporting clinical or preclinical results published in the last 20 years (1998–2018) and dealing with the etiology of ICTEV. Exclusion criteria were articles written in other languages or studies with a focus on secondary/syndromic CTEV, such as distal arthrogryposis, myelomeningocele, and Moebius syndrome. We also excluded all the remaining duplicates, articles dealing with other topics, with poor scientific methodology, or without an accessible abstract.

**Fig. 1:**
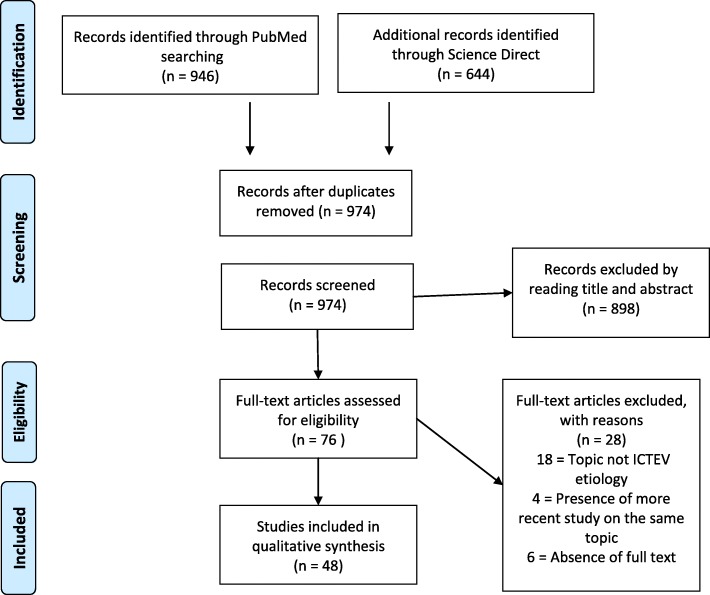
PRISMA (Preferred Reporting Items for Systematic Reviews and Meta-Analysis) flowchart of the systematic literature review

At the end of the first screening, we selected *n* = 76 articles that were eligible for full-text reading. After reading the full text, we ultimately selected *n* = 48 articles that satisfy the criteria. A PRISMA [16] flowchart of the selection and screening method is provided in Fig. 1. Reference lists from the selected papers were also screened.

## Results

The included articles [3, 4, 12, 17–62] mainly focus on genetic research [17, 21–23, 25, 27, 29–31, 37–50, 52, 54–56, 58, 60, 61], epidemiological studies [12, 19, 20, 24, 32, 33, 35, 36, 53], MRI analysis [26, 51, 59], and histological histochemical analysis [18, 24, 28, 57] in ICTEV patients. Two previous reviews [3, 4, 62] reporting a significant analysis of the current evidence and research prospective were also included since they are part of the present evidence of the topic. The main findings of the included articles except for the two reviews were summarized (Table 1.)

**Table 1:** Main findings of the included article

Ref	Author	Subjects	Pathway/molecule involved	Results
[12]	Kruse et al. (2008)	1093 individuals: 291 with clubfoot and 802 unaffected relatives	Polygenic threshold model with sex dimorphism	This study demonstrates the presence of the Carter effect in idiopathic clubfoot. A polygenic inheritance of clubfoot can explain this effect, with females requiring a greater genetic load to be affected.
[17]	Gurnett et al. (2008)	A five-generation family with asymmetric right-sided predominant idiopathic clubfoot	*PITX1*	A single missense mutation (c.388G → A) was identified in *PITX1 through Genome-wide linkage analysis.*
[18]	Poon (2009)	Primary cell culturefrom the medial aspect of the talonavicular joint and from the plantar surface of the calcaneocuboid joint (around 10 ft)	Beta-catenin	There was a more than twofold increase in the beta-catenin protein in the contracted tissues.
[19]	Pagnotta et al. (2011)	Three homozygous preterm triplets	Presence of Bilateral ICTEV	Such a presentation had not been previously described and supports a genetic etiology of congenital idiopathic talipes equinovarus deformity.
[20]	Sahin et al. (2013)	28 cases (infants with idiopathic CTEV) and 575 controls (healthy infants) were recruited	Case-control study	Significant risk factors for idiopathic CTEV were work status (employed), consanguineous marriage, sex (male), and gestational age (> 42 weeks).
[21]	Alvarado et al.(2013)	413 isolated talipes equinovarus patients	*PITX1*, *TBX4*, *HOXC13*, *UTX*, *CHD1*, and *RIPPLY2*	A genome-wide screening found 12 rare copy number variants segregated with talipes equinovarus in multiplex pedigrees, containing the developmentally expressed transcription factors and transcriptional regulators *PITX1, TBX4, HOXC13, UTX, CHD* (chromodomain protein)*1, and RIPPLY2*
[22]	Shyy et al. (2009)	CAND2 gene was sequenced in 256 clubfoot patients, and 75 control patients, while WNT7a was screened using 56 clubfoot patients and 50 control patients	CAND2 and WNT7	Polymorphism was found in each gene, but the single nucleotide change in CAND2 was a silent mutation that did not alter the amino acid product, and the single nucleotide change in WNT7a was in the upstream, non-coding or promoter region before the start codon.
[23]	Shyy et al. (2010)	24 bilateral congenital idiopathic clubfoot patients and 24 matched controls and screened an additional 76 patients in each discovered SNP	MYH 1, 2, 3, and 8	Many single-nucleotide polymorphisms were found; none proved to be significantly associated with the phenotype of congenital idiopathic clubfoot.
[24]	Herceg et al. (2006)	95 ft in 68 ICTEV patients yielded a total of 431 muscle specimens	Histological and histochemical muscle specimens analysis	This study does not support the theory that a neuromuscular abnormality may be significant in the etiology of idiopathic talipes equinovarus because of the absence of significant alteration.
[25]	Wang et al. (2008)	Rat embryo	HOXD13 – FHL1	The findings suggest that HOXD13 may regulate the expression of FHL1 in the development of ICTEV.
[26]	Ippolito et al. (2009)	MR of both legs was taken in three cohorts of patients with unilateral ICTEV: 8 untreated new-borns (age range 10 days to 2 weeks); 8 children who had been treated with the Ponseti method (age range 2–4 years); 8 adults whose deformity had been corrected (age range 19–23 years)	Muscular atrophy	The study shows that leg muscular atrophy is a primitive pathological component of CCF which is already present in the early stages of fetal CCF development.
[27]	Hecht et al. (2007)	56 multiplex ITEV families, 57 trios with a positive family history and 160 simplex trios with ITEV	NAT 1 and NAT 2	The result suggests that slow NAT2 acetylation may be a risk factor for ITEV.
[28]	Ošt’ádal et al. (2015)	13 relapsed ICTEV	Proteomic analysis of the extracellular matrix	The major result of the present study was the observation that the extracellular matrix in clubfoot is composed of an additional 16 proteins, including collagens V, VI, and XII, as well as the previously described collagen types I and III and transforming growth factor beta.
[29]	Gurnett et al. (2009)	31 patients (five with familial vertical talus, 20 with familial clubfoot, and six with DA1	TNNT3, MYH3, TPM2	Although mutations in MYH3, TNNT3, and TPM2 are frequently associated with distal arthrogryposis syndromes, they were not present in patients with familial vertical talus or clubfoot.
[30]	Wang et al. (2013)	Abductor hallucis muscle samples were obtained from 15 ICTEV patients. Peripheral blood samples were obtained from 84 ICTEV patients	SOX 9	mRNA and protein expression levels of SOX9 were detected through real-time polymerase chain reaction and western blot analysis, respectively and were found to be significantly higher in ICTEV muscle samples compared with those in control samples
[31]	Cao et al. (2009)	Rat ICTEV model	HOXD13 – Gli3	Findings suggest that *HoxD13* directly interacts with the promoter of *Gli3*. The increase of *Gli3* expression in the ICTEV model animal might result from the low expression of *HoxD13.*
[32]	Engell et al. (2014)	46,418 twin individuals	Twin study	The study found an overall self-reported prevalence of congenital clubfoot of 0.0027. They concluded that non-genetic factors must play a role, and a genetic factor might contribute, in the etiology of congenital clubfoot.
[33]	Parker et al. (2009)	6139 cases of clubfoot from 2001 through 2005 plus 10 controls per case.	Risk factors and prevalence	The overall prevalence of clubfoot was 1.29 per 1000 in live births. Maternal smoking and diabetes showed significant associations.
[34]	Hackshaw et al. (2011)	15,673 clubfoot cases	Smoking	Significant positive associations with maternal smoking were found for clubfoot (OR 1.28, 95% CI 1.10–1.47)
[35]	Dickinson et al (2008)	443 cases of clubfoot and 4492 randomly sampled controls	Smoking	This study is consistent with the hypothesis that smoking during pregnancy is associated with a slightly increased risk of an infant being born with clubfoot.
[36]	Honein (2000)	346 infants with isolated clubfoot and 3029 infants without defects	Smoking	This study confirms the importance of familial factors and smoking in the etiology of clubfoot and identifies a potentially important interaction.
[37]	Wang et al. (2005)	84 idiopathic congenital talipes equinovarus nuclear pedigrees	HOXD10, HOXD12 and HOXD13	HOXD12 andHOXD13 are important susceptible genes of idiopathic congenital talipes equinovarus.
[38]	Ester et al. (2009)	179 extended families and 331 simplex families and 88 trios with a positive family history. The validation population consisted of 144 NHW simplex trios	HOXA and HOXD gene clusters	These results suggest a biologic model for clubfoot in which perturbation of HOX and apoptotic genes together affect muscle and limb development, which may cause the downstream failure of limb rotation into a plantar grade position.
[39]	Alvarado et al. (2016)	1178 probands with clubfoot or verticaltalus and 1775 controls	HOXC	Since HOXD10 has been implicated in the etiology of congenital vertical talus, variation in its expression may contribute to the lower limb phenotypes occurring with 5’ HOXC microdeletions.
[40]	Weymouth et al. (2016)	Nuclear extracts isolated from undifferentiated and differentiated C2C12 mouse muscle cells	HOXA9, TPM1, and TPM2	Results show that associated promoter variants in HOXA9, TPM1, and TPM2, alter promoter expression suggesting that they have a functional role.
[41]	Liu et al. (2011)	25 children with ICTEV and 5 normal controls	COL9A1	COL9A1 protein is highly expressed in patients with ICTEV and rs1135056, which is located in the coding region of COL9A1 gene, may be associated with the pathogenesis of ICTEV.
[42]	Zhao et al. (2016)	87 children with congenital talipes equinovarus and 174 control subjects	COL9A1	In conclusion, our results indicate that the COL9A1 rs35470562 variant may contribute to congenital talipes equinovarus susceptibility in the Chinese population examined.
[43]	Zhang et al. (2006)	84 idiopathic congenital talipes equinovarus nuclear pedigree	GLI3	There is an association between GLI3 gene and ICTEV, and exons 9,10,11,12 are not its mutation hot spots.
[44]	Lu et al. (2012)	605 probands (from 148 multiplex and 457 simplex families) with non-syndromic clubfoot	*TBX4* and chromosome 17q23.1q23.2	These results demonstrate that variation in and around the *TBX4* gene and the 17q23.1q23.2 microduplication are not a frequent cause of this common orthopedic birth defect and narrows the 17q23.1q23.2 non-syndromic clubfoot-associated region.
[45]	Peterson et al. (2014)	One family: mother, daughter, and two sons	TBX4	Although TBX4 remains the candidate gene for congenital clubfoot involving 17q23.1–q23.2 duplications, the explanation for variable expressivity and penetrance remains unknown.
[46]	Alvarado et al. (2011)	Mice model	Pitx1	Morphological data suggest that PITX1 haplo insufficiency may cause a developmental field defect preferentially affecting the lateral lower leg, a theory that accounts for similar findings in human clubfoot.
[47]	Alvarado et al. (2010)	66 isolated idiopathic clubfoot probands with at least one affected first-degree relative	TBX4	Our result suggests that this chromosome 17q23.1q23.2 microduplication is a relatively common cause of familial isolated clubfoot and provides strong evidence linking clubfoot etiology to abnormal early limb development.
[48]	Dobbs et al. (2006)	21 affected individuals and 17 unaffected individuals	HOXD10	This mutation was recently described in a family of Italian descent with congenital vertical talus (CVT) and Charcot-Marie-Tooth deformity HOXD10 gene mutations were not identified in any of the other families or sporadic patients with CVT, suggesting that genetic heterogeneity underlies this disorder.
[49]	Shrimpton et al. (2004)	36 members of a single family	*HOXD10*	In the study family, this mutation was fully penetrant and exhibited significant evidence of linkage (LOD 6.33; *θ* = 0), and it very likely accounts for congenital vertical talus in heterozygotes.
[50]	Heck et al. (2005)	57 multiplex ITEV families and 83 simplex trios	CASP8, CASP10	Genotyping of SNPs throughout the genes in this sample of ITEV families has revealed positive linkage with association to the major allele of a variant in CASP10 in simplex ITEV white and Hispanic trios.
[51]	Duce et al. (2013)	The lower legs of six CTEV (2 bilateral, 4 unilateral) and five control young adults (ages 12–28)	3D MRI and MRA	The proportion of muscle in affected CTEV legs was significantly reduced compared with control and unaffected CTEV legs, while proportion of muscular fat increased. No spatial abnormalities in the location or branching of arteries were detected, but hypoplastic anomalies were observed.
[52]	Zhang et al. (2016)	29 individuals of the same family	ANXA3 and MTHFR	Following whole genome sequencing and comparative analysis, several differential gene variants were identified to enable a further distinction from clubfoot.
[53]	Lochmiller et al. (1998)	A total of 285 propositi were ascertained, with detailed family history information available in 173 cases and medical records on the remaining 112 propositi	Genetic and environmental risk factor	A family history of ITEV was noted in 24.4% of all propositi studied. These findings, in addition to the detailed analysis of 53 pedigrees with ITEV history, suggest that the potential role of a gene or genes operating in high-risk families produces this foot deformity.
[54]	Yang et al. (2016)	three-generation pedigree and 53 sporadic patients with CTEV	*FLNB*	The results provide evidence for the involvement of FLNB in the pathogenesis of isolated CTEV and have expanded the clinical spectrum of FLNB mutations.
[55]	Zhang et al. (2014)	96 isolated clubfoot patients and 1000 controls	NCOR2, ZNF664 FOXN3, SORCS1, and MMP7/TMEM123	The study suggests a potential role for common genetic variation in several genes that have not previously been implicated in clubfoot pathogenesis.
[56]	Weymouth et al. (2011)	The discovery dataset was comprised of 224 multiplex families, which include 137 non Hispanic white (NHW) and 87 Hispanic families, and 357 simplex families, which includes 139 NHW and 218 Hispanic families	TNNC2 and TPM1	The results reported suggest that variation in genes that encode contractile proteins of skeletal myofibers may play a role in the etiology of clubfoot.
[57]	Gilbert et al. (2001)	Two normal feet from a 40-week-old stillborn fetus, and samples from six calcanei from children with relapsed CTEV, aged 2, 3, 4, and 5 years, were studied	Histological analysis of the calcaneum	The process of ossification in CTEV was retarded. The talipes cartilage matrix contained fewer cartilage canals and chondrocytes
[58]	Ester et al. (2007)	210 simplex trios and 139 multiplex families	SNPs spanning seven apoptotic genes-Casp3, Casp8, Casp9, Casp10, Bid, Bcl-2, and Apaf1	One SNP in each of the genes provided impressive evidence of association with idiopathic talipes equinovarus
[60]	Sharp et al (2006).	375 case-parent triads	C677T polymorphism in MTHFR	DNA synthesis may be relevant in clubfoot development
[61]	Bonafe et al. (2002)	125 ITEV probands and their parents	DTDST	The R279W mutation is no more frequent in this population of ITEV probands than in controls.

ICTEV has historically been linked to several risk factors: oligohydramnios, smoking, parental age, parental education, parity, maternal anxiety or depression, alcohol use, and season of birth. A previous epidemiological study based on the difference in prevalence in different communities suggested that environmental factors have a role in pathogenesis. In 2014, a twin study done in Denmark surveyed 34,485 twins and found evidence of a role of environmental factors. The authors concluded that the presence of a genetic role in the development of the disease was not enough to explain the results. Therefore, they reported strong evidence of the presence of environmental factors to explain the statistical analysis [32]. Another study from 2013 [20] was conducted in a rural area of Turkey and showed that parental consanguineous marriage was associated with a higher risk of ICTEV. Even though the sample investigated was small, this result may support an etiology based on multiple genes and environmental factors.

Even though the role of environmental factors has been confirmed by several studies, all the proposed factors except for smoking were not significantly associated with ICTEV, which was linked to DNA oxidative damage caused by tobacco smoking [33–36]. A meta-analysis examined 172 reports containing cumulative data for 173,687 individuals and 11,674,332 unaffected controls published from 1959 to 2010. The analysis looked at the effects of smoking during pregnancy and showed that 15,673 individuals with CTEV and maternal smoking had an OR of 1.28 (95% CI 1.10–1.47) [34].

Genes involved in the metabolism of smoke-derived products may also contribute to the development of birth defects. Therefore, N-acetylation genes including NAT1, NAT2, and other related genes were screened for association analysis. Polymorphisms in the NAT2 gene cause decreased acetylation activity and have been associated with TEV. This suggests a deficit in the biotransformation of aromatic amines and the accumulation of DNA adducts, leading to a potential toxic effect and the development of TEV [27].

Hecht et al. [27] examined the variants of the NAT2 gene in 56 ICTEV multiplex families, 57 trios with a positive family history, and 160 simplex individuals. They reported a slight decrease in the expected number of homozygotes for the normal NAT2 allele in the Hispanic simplex trios. Significantly, a slow NAT2 acetylator phenotype was detected among the ICTEV patients, suggesting that slow acetylation may be a risk factor for ICTEV.

### Genetic factors

Genetics has a crucial role in the development of ICTEV, even though no major gene candidate has been identified [3]. There is evidence of a family history of TEV in 24–50% of cases [22]. Results from twin studies showed concordance in monozygotic twins (32%) compared to dizygotic twins (2.9%), and a frequency of recurrence in 10–20% of families supports a role for genes in ICTEV [19, 32, 63]. There is also reported a unique case of bilateral ICTEV in preterm triplets, which provides even further support for a genetic etiology [19]. Many different families of genes were identified to play a role in the disease and a prospective role in the development of personalized conservative and surgical approaches [64]. Several families of genes and pathways were identified and investigated using mainly the candidate gene approach.

### Homeobox family genes

The homeobox genes represent a family of transcription factors that play a central role in the morphogenesis processes of embryonic development. In particular, this family determines the correct genesis of the axial skeleton and limbs, which is why they were proposed as candidate genes for ICTEV pathogenesis [65]. Several candidate gene studies found a locus of genetic susceptibility associated with ICTEV in the HOX domain and the caspase domain [25, 37].

In 2009 and 2016, two large studies showed that ICTEV was associated with alteration in the regulator domain of HoxA and HoxD [38, 39]. Higher activity of the promotor was also reported as a result of promotor variation [40]. Based on the emerging evidence, we can assume that perturbation to the HOXA, HOXC, and HOXD clusters of genes may play a role in the etiology and pathogenesis of ICTEV [66].

### Caspases pathway genes

Cysteine-dependent aspartate-directed proteases (caspases) are part of a family of cysteine proteases that play essential roles in apoptosis, necrosis, and inflammation processes. This family was investigated since caspase activity seems to be related to correct limb development, and related genes were first associated with ICTEV in 2005 by Heck et al. [50].

A CASP10 gene variant was found in simplex ICTEV in white and Hispanic trios. In 2007, Ester et al. [58] researched other alterations in three caspase genes. They genotyped SNPs of three different genes (Casp8, Casp10, and CFLAR) to investigate their association with ICTEV. One SNP in each of the genes was associated with the disease. Several haplotypes constructed from these SNPs displayed altered transmission, suggesting that genetic variation in apoptotic genes may play a role in the development of ICTEV.

### Collagen family genes

The collagen family genes were also linked to ICTEV. The focus of related genetic research has been on the COL9A1 and COL1A1 genes. COL9A1 encodes for one of the three alpha chains of type IX collagen, a component of the hyaline cartilage, while COL1A1 encodes for pro-alpha 1 chains of type I collagen, a component of most connective tissue that is abundant in bone and tendons. In 2008, COL1A1 was investigated in healthy and ICTEV patients. The study reported a higher expression of COL1A1 in patients with ICTEV than in healthy patients. A − 161(T → C) heterozygous mutation and a + 274(C → G) homozygous mutation were also identified in the COL1A1 gene in patients with ICTEV, suggesting that COL1A1 variants could be linked to the onset of ICTEV [37].

Based on previous studies, Wang et al. [30] investigated genes that regulate COL91A1 expression (SOX9) in 2012. They reported no mutations of the gene but a higher expression of SOX9 in the muscular cells of ICTEV patients. COL9A1 polymorphism seems to modulate the gene expression and influence the protein function. Three studies reported a role of these polymorphisms in ICTEV in the populations examined [41, 42, 67].

### GLI3 gene

The GLI3 gene encodes for a C2H2-type zinc finger protein of the GLI family. In 2005, a study showed how a mutation of this gene was associated with the occurrence of ICTEV [43]. In 2009, another study [31] reported how HoxD13 directly interacts with the promoter of GLI3. They observed that GLI3 mRNA and protein expression levels were increased in ICTEV-model rats. This may mean that HOXD13 is a transcription factor of GLI3. Low expression of HOXD13 might lead to increased GLI3 expression level during limb formation, which likely plays a key role in ICTEV pathogenesis.

### T-box family

The T-box family comprises transcription factors that play a crucial role in embryogenesis and morphogenesis. Like other genes with a similar role, they are candidates for possible genetics inducers of ICTEV. TBX3 and TBX4 are the main family members studied. The TBX3 protein is a transcriptional factor of the T-box family. A 2014 study reported that mutations in this gene affect limb development were proven to have transmission disequilibrium in ICTEV patients, suggesting susceptibility to ICTEV [65].

### PITX1-TBX4 pathway

TBX4 protein is a transcriptional factor that is mainly expressed in the hindlimb and is thus associated with ICTEV pathogenesis [68]. It was further studied in association with another transcriptional factor, PITX1, which is part of the same pathway. The PITX1-TBX4 pathway is responsible for early limb development. Numerous studies report that mutations in the genes encoding the transcription factors PITX1 and TBX4 lead to a reduction in lower-limb musculature and classic clubfoot phenotypes in both humans and mice [17, 44–46]. Studies support a role of the PITX1-TBX4 developmental pathway in TEV etiology.

Gurnett et al. [17] researched these pathway alterations in a five-generation family with asymmetric ICTEV segregating as an autosomal dominant condition. A single missense mutation (E130K) located in a highly conserved domain of the PITX1 gene has been identified. Another study showed that PITX1 downregulation causes a clubfoot-like phenotype in mice, thus providing evidence of the involvement of PITX in ICTEV pathogenesis [46].

TBX4 microdeletions and microduplications have been reported in patients affected by ICTEV, suggesting that chromosome 17q23.1q23.2 microduplication is a relatively common cause of familial isolated clubfoot [47]. However, in 2012, Lu et al. [44] examined the possible correlation between the hindfoot-specific gene TBX4 and ICTEV. They concluded that the microduplication is a rare cause of familial isolated clubfoot and can be segregated as an autosomal dominant phenotype. Significant variations were not present in the two known TBX4 hindlimb enhancers sequenced in 95 patients from simplex families.

A recent study conducted in 2017 reported that the PITX1-TBX4 pathway can be associated with HOXC alteration in vertical talus. They identified a HOXC13 deletion that segregated with clubfoot in a three-generation family [21]. Deletions of part of the HOXC gene cluster were later identified in two of five families with autosomal dominant isolated congenital vertical talus, suggesting that it is a possible cause of familial vertical talus [39]. Interestingly, HOXD10 mutations were previously identified in two families with vertical talus [48, 49], which strongly supports a role of homeobox gene mutations in the etiology of isolated vertical talus. However, because mutations in the PITX1-TBX4-HOXC pathway are infrequent in patients with clubfoot, other genetic mechanisms remain to be discovered and investigated [64].

### Troponin and tropomyosin genes

The troponin (Tn) family is a protein complex involved in striated muscle contraction and has three subunits: Tn-I, Tn-T, and Tn-C. The Tn-I subunit inhibits actomyosin ATPase, while the Tn-T subunit binds tropomyosin and Tn-C. The Tn-C subunit binds calcium and overcomes the inhibitory action of the troponin complex on actin filaments.

A 2011 study analyzed 15 genes encoding proteins that control myofiber contractility in a cohort of both non-Hispanic white (NHW) and Hispanic families. They reported an association between ICTEV patients and multiple SNPs of two genes regulating troponin activity, TNNC2 and TPM1, suggesting a possible role in the etiology [56].

TPM1 is a member of the tropomyosin family, which comprises actin-binding proteins involved in the contraction of both striated and smooth muscles and the cytoskeleton of non-muscular cells. The associations of multiple SNPs in the TPM1 gene with ICTEV suggest a potential role of genes that encode contractile proteins of skeletal myofibers in the etiology of ICTEV [23]. ICTEV patients present a clinically evident alteration of the calf muscle at birth, which usually resituates after treatment [26, 69, 70]. This suggests the involvement of genes that play a role in muscle morphogenesis.

Distal arthrogryposis is a cause of syndromic TEV that is characterized by variations in genes that encode for components of the muscle contractile complex (MYH3, TPM2, TNNT3, TNNI2, and MYH8), resulting in muscle contractures. The similar phenotype suggests that these genes could be candidate genes. However, one study found that the development of the disease was different in ICTEV and in DA, even though it suggested a potential role of many regulatory candidate genes that could cause developmental defects in the hypaxial musculature that is invariably observed in clubfoot [24].

In contrast to other studies, Gurnett et al. [29] investigated 39 patients in 2009 to find mutations in the TNNT3, MYH3, and TPM2 genes in patients with ICTEV. The results showed an absence of correlation of these mutations in ICTEV patients. Recent evidence showed an absence of significant histological and cytological alteration of muscles after treatment [24]. Another work proposed an innovative 3D RM study of the muscle morphology to show how intramuscular fat distribution plays an important role in the morphology of the leg [51]. The potential of using MRI has also been suggested to better understand the clinical severity of an affected patient [71].

### CAND2 and Wnt7a

In 2009, a study investigated two candidate genes, CAND2 and Wnt7a, and tested their role in the pathogenesis of ICTEV. They genotyped the CAND2 gene in 256 clubfoot patients and 75 control patients, while Wnt7a was screened using 56 clubfoot patients and 50 control patients. The study reported a polymorphism in each gene. However, the association results indicated that CAND2 and WNT7a are not major genes involved in the etiology of ICTEV [22].

In 2009, Poon et al. [18] showed that foot tissues were related to higher beta catenin levels. This was probably related to the Wnt signaling pathway and the synthesis of type III collagen. In particular, a higher amount of type III collagen was reported in studies analyzing the extracellular matrix of ICTEV tissues [28, 57]. More research is needed to understand the interactions of these growth factors with other proteins and their role in ICTEV etiology.

### Dysplasia sulfate transporter gene

The dysplasia sulfate transporter (DTDST) gene was suggested to cause ICTEV and investigated by Bonafé et al. [61]. They tested whether R279 W mutations are responsible for the occurrence, but alterations in the coding region were not identified in 10 probands with ICTEV and a positive family history. The authors concluded that the R279 W mutation is no more frequent in this population of ICTEV probands than in controls.

### Methylenetetrahydrofolate reductase gene

In 2006, Sharp et al. found that children who carry the 677T variant of the methylenetetrahydrofolate reductase gene (MTHFR) have a lower risk of ICTEV [60]. Another study later used whole genome sequencing to investigate the variants of MTHFR and the annexin A3 gene (ANXA3). They reported an MTHFR variant that is different from the variant associated with clubfoot in the study by Sharp et al. [52]. Bioinformatic analysis showed that the protein-binding region could be altered by this mutation (a sequence shift: the wild type is 264, while the mutant type is 267). Despite sharing some similar symptoms, these findings imply that the variant was associated with another genetic disease and not ICTEV. Furthermore, specific CNV profiles were identified in association with the diseased samples, thus further demonstrating the complexity of this multigenerational disorder [52].

## Discussion

The etiology of ICTEV remains unknown as stated in recent reviews [3, 4, 72]. Many theories have been developed, but no one has clarified the major roles in the pathogenesis of idiopathic clubfoot. Recent studies have focused on the interaction between genetics and environmental factors, showing a multifactorial identity of the disease. Today, this remains the most validated theory.

A recent paper [73] reported a genetic analysis on a spontaneous autosomal recessive mouse model of peroneal muscular atrophy (PMA). It was used to understand the underlying developmental causes of ICTEV. The PMA mutation was mapped, and several candidate genes were identified, of which LIMK1 was upregulated in mutant mice. Collison et al. also reported that in chickens, LIMK1 upregulation can cause sciatic nerve defects and a TEV phenotype [73]. Further studies should be conducted using these models.

The years of research using the candidate gene approach has provided us more knowledge on the possible pathways involved in ICTEV pathogenesis, but it has failed to find a major gene causing the disease. The literature illustrates the great heterogeneity of the genetic causes of ICTEV. The candidate approach has probably not recognized the real amount of various causative variants and has likely underestimated the phenotypical and genotypical variants. The reported studies were also done using also different technical approaches, such as genome-wide association analysis (GWAS), linkage analysis, the technique of copy number variation, and whole exome sequencing. These next-generation genetic analyses should lead future studies on ICTEV etiology. Collaborative multicenter studies involving large populations might be a necessary step to shed light on the etiology of this complex disease. ICTEV inheritance is most often considered complex, with more than 75% of all cases reporting no family history [17, 53]. Thus, a large-scale GWAS study might reveal interesting results.

The filamin B (FLNB) gene encodes a member of the filamin family. The encoded protein interacts with glycoprotein Ib alpha as part of the process of repairing vascular injuries. The platelet glycoprotein Ib complex includes glycoprotein Ib alpha and binds the actin cytoskeleton. In 2016, Yang et al. performed WES sequencing and Sanger sequencing to identify and validate disease-causing mutations in a three-generation pedigree and 53 sporadic patients with ICTEV, respectively. A c.4717G>T (p.D1573Y) mutation in the FLNB gene, which co-segregated with ICETV, was identified in the pedigree. Two additional novel missense mutations in the same gene, c.1897A>G (p.M633V) and c.2195A>G (p.Y732C), were identified in the 53 sporadic patients, thus providing evidence of the involvement of the FLNB gene in ICTEV [54].

In 2014, Zhang et al. performed a GWAS study of the DNA of 396 isolated clubfoot patients and 1000 controls of European descent. The DNA was genotyped for > 600,000 single nucleotide polymorphisms (SNPs) to identify novel genes for ICTEV. The variants selected were then replicated with an independent cohort of 370 isolated clubfoot cases and 363 controls of European descent. The study found a strong association with the disease for an intergenic SNP on chromosome 12q24.31 between NCOR2 and ZNF664 (rs7969148, OR = 0.58, *p* = 1.25 × 10^–5^), which was significant on replication (combined OR = 0.63, *p* = 1.90 × 10^–7^). However, additional suggestive SNPs (Hox Genes, PITX1, TBX4, FOXN3, SORCS1, and MMP7/TMEM123) in the identified pathways were not significant in the replication phase [55]. With the aid of a new animal model, next-generation studies may have the potential to identify genes underlying the phenotype and elucidate the inheritance pattern and penetrance of the disorder [3].

## Conclusions

The available literature on the etiology of ICTEV presents major limitations in terms of great heterogeneity and lack of high-profile studies. Although many studies have focused on the genetic background of the disease, there is a lack of consensus on one or multiple targets. Recent evidence shows a major role of both genetic and environmental factors. Thus far, smoking is the major environmental factor supported by recent evidence. The etiology of ICTEV is probably multifactorial and associated with multiple gene alterations, and large multi-center studies are required to investigate them. Further large international collaborative studies using next-generation sequencing technology in ICTEV patients are strongly encouraged.

## Abbreviations

DTDST: Dysplasia sulphate transporter
FLNB: Filamin B
GWAS: Genome wide association analysis
ICTEV: Idiopathic congenital talipes equinovarus
MTHFR: Methylenetetrahydrofolate reductase
PMA: Peroneal muscular atrophy
PRISMA: Preferred Reporting Items for Systematic Reviews and Meta-Analyses
